# Epigenetic Basis of Cellular Senescence and Its Implications in Aging

**DOI:** 10.3390/genes8120343

**Published:** 2017-11-24

**Authors:** Timothy Nacarelli, Pingyu Liu, Rugang Zhang

**Affiliations:** Gene Expression and Regulation Program, The Wistar Institute, Philadelphia, PA 19104, USA; piliu@wistar.org (P.L.); rzhang@wistar.org (R.Z.)

**Keywords:** cellular senescence, aging, epigenetic change, SAHF, SASP

## Abstract

Cellular senescence is a tumor suppressive response that has become recognized as a major contributor of tissue aging. Senescent cells undergo a stable proliferative arrest that protects against neoplastic transformation, but acquire a secretory phenotype that has long-term deleterious effects. Studies are still unraveling the effector mechanisms that underlie these senescence responses with the goal to identify therapeutic interventions. Such effector mechanisms have been linked to the dramatic remodeling in the epigenetic and chromatin landscape that accompany cellular senescence. We discuss these senescence-associated epigenetic changes and their impact on the senescence phenotypes, notably the proliferative arrest and senescence associated secretory phenotype (SASP). We also explore possible epigenetic targets to suppress the deleterious effects of senescent cells that contribute towards aging.

## 1. Cellular Senescence: A Double-Edged Sword against Cancer

Cellular senescence was originally identified as a cellular aging phenomenon, but is now recognized as an intrinsic tumor suppressive mechanism that is accompanied by distinct phenotypic changes, such as epigenetic and chromatin remodeling of nuclear architecture. Hayflick and Moorhead first identified cellular senescence when they found that primary human fibroblasts undergo a finite number of divisions before entering a stable proliferative arrest in culture, now termed replicative senescence [1,2]. Replicative senescence was eventually identified as a consequence of telomere attrition following repeated cell divisions, which is thought to reflect aging at the cellular level [3,4,5]. Cellular senescence was shown to apply in vivo with tissue aging after senescent cells were identified in aged tissue and at sites of age-related pathologies [6]. Cellular senescence can also be induced by a variety of potentially tumorigenic stimuli, including genotoxic stress, oncogene activation, and oxidative stress. These stressors cause persistent DNA damage and activate the DNA damage response [7]. Besides DNA damage, studies have found other stressors can initiate cellular senescence, such as in the case of metabolic stress-induced senescence [8]. Studies have indicated cellular senescence as a barrier for tumorigenesis in vivo by the presence of senescent cells at pre-malignant lesions, but not at malignant tumors [9]. Collectively, these findings established the hypothesis that cellular senescence serves a tumor suppressive role and prevents the proliferation of stressed cells harboring oncogenic potential.

Cellular senescence has long-term deleterious effects that mediate tissue aging, despite mounting a protective tumor suppressive response. Senescent cells are thought to exert these deleterious effects through cell-autonomous and non-autonomous mechanisms. To avoid neoplastic transformation, senescent cells undergo a cell-autonomous proliferative arrest, which is maintained by the tumor suppressive p53 and cyclin dependent kinases 4 and 6 (CDK4/CDK6) inhibitor p16 (p16^INK4A^) pathways. The p53 pathway is predominantly activated through the DNA damage response following genomic damage, including double strand DNA breaks and telomere dysfunction. DNA damage is sensed through the ataxia-telangiectasia mutated (ATM) kinase that signals to stabilize and activate p53. p53 relays the signal of genomic stress by transcriptionally upregulating p21^CIP1/WAF1^ [10,11]. p21^CIP1/WAF1^ functions in concert with p16^INK4A^ to promote hypophosphorylation of retinoblastoma and stably arrest the cell cycle at the G1 phase [12]. Unlike p21, studies have not identified a principal activator of p16^INK4A^ but found that it is activated by several stress-related pathways, including the p38 MAP kinase pathway [13]. Under the growth-arrested state, senescent cells resist cell death and persist for prolonged periods of time [14]. Consequently, senescent cells accumulate in tissue and are suspected of exhausting tissue of proliferation-competent cells and renewable stem cells over time, diminishing homeostasis and regenerative capacity of the tissue [15,16,17]. This idea is supported by evidence that p16^INK4A^ expression is elevated and associated with reduced regeneration in multiple stem cell compartments in mice, including in the bone marrow, pancreas, and brain [18,19,20]. Senescent cells can also disrupt tissue homeostasis in a cell-non-autonomous fashion by acquiring a senescence-associated secretory phenotype (SASP). The SASP encompasses a wide range of factors that promote different aspects of the senescence phenotype. These factors include pro-inflammatory cytokines and chemokines, proangiogenic factors such as matrix metalloproteinases and vascular endothelial growth factor (VEGF), and other soluble factors that assist the senescence phenotype, including plasminogen activator inhibitor and prostaglandin E2 [21]. The SASP has been shown to have autocrine and paracrine effects. For instance, interleukin (IL)-6 and 8 have been shown to function in an autocrine manner to promote the DNA damage response and proliferative arrest of senescent cells [22,23,24]. Additionally, transforming growth factor beta has been recently shown to promote cellular senescence of neighboring cells [25]. However, the most profound cell non-autonomous effects are mediated by paracrine signaling that stimulates chronic inflammation [26].

Chronic tissue inflammation is not only a major contributor of age-related degeneration, but also cancer phenotypes by creating a pro-tumorigenic microenvironment [7,26,27]. Thus, targeting the proinflammatory effects of senescent cells is a strategy to suppress the aging process and the development of a myriad of pathologies. The deleterious role senescent cells play during aging and disease was demonstrated using transgenic *INK-ATTAC* mice that allow for the selective elimination of senescent cells, which entailed apoptosis induction of high p16^INK4A^ expressing cells. Using this system, studies showed that selectively eliminating senescent cells improved the healthspan and suppressed the pathologic features of progeriod and naturally-aged mice [15,28]. The success of these studies led many to research pharmacological drugs that induce apoptosis of senescent cells, which has been termed senolytic compounds [16]. Such senolytic compounds have been shown to eliminate senescent cells by inhibiting resistance to apoptosis that is integral to the program of cellular senescence [29,30]. However, not all senescent cell types respond to senolytic compounds, which has been found in the case of senescent preadipocytes [31]. Although the selectivity for senescent cells is still being improved, studies using senolytic compounds have shown overall positive effects on attenuating disease pathology and restoring tissue function [32,33].

Senescent cells undergo distinct phenotypic changes, including an enlargement in cellular morphology and elevated lysosomal β-galactosidase activity, which is detected by histochemical staining [34,35]. It is important to note that cellular senescence is not identified by a single characteristic, but a combination of markers with the most common being proliferation arrest and elevated p16 expression and β-galactosidase activity [16]. Thus, the lack of a single universal marker of cellular senescence has posed an obstacle for the detection of senescent cells in vivo. Senescent cells also undergo nuclear phenotypic changes wherein the epigenetic and chromatin landscape undergo widespread alterations. Such changes have been linked to the altered gene expression and effector mechanisms that play a role in the proliferative arrest and acquisition of the SASP during cellular senescence. Recent studies have indicated the prospect of therapeutically targeting epigenetic changes to suppress the deleterious effects of senescent cells. Here, we will review these possibilities and the impact of chromatin and epigenetic changes in regulating cellular senescence and susceptibility for aging.

## 2. Epigenetic Changes during Cellular Senescence and Aging

### 2.1. Heterochromatin Changes Accompany Cellular Senescence

The most striking nuclear phenotype of cellular senescence is the formation of facultative heterochromatin domains, termed senescence-associated heterochromatic foci (SAHF). The SAHF are easily visible following diamidino-2-phenylindole (DAPI) staining as distinct DNA foci [36]. The foci reflect compacted chromatin and are enriched in a variety of heterochromatic markers, such as hypoacetylated histones, histone H3 lysine 9 and 27 trimethylation (H3K9me3 and H3K27me3), heterochromatin protein 1 (HP1) family proteins, and the histone variant macroH2A. These epigenetic marks repress the transcription of key proliferation-related genes, linking SAHF to the tumor suppressive proliferative arrest of senescent cells [36,37]. The SAHF can also recruit effectors to aid in the senescence arrest, which has found to be the case in the recruitment of the chromatin remodeling enzyme ATRX by H3K9me3 and HP1γ following therapy-induced senescence [38]. Several lines of evidence indicate the appearance of these epigenetic marks that reflect the SAHF during tissue aging and cellular senescence in vivo [17,39,40,41,42]. The SAHF has additional roles in addition to supporting cell cycle arrest during cellular senescence. For instance, the SAHF has been shown to protect oncogene-induced senescent cells from apoptosis by dampening the DNA damage response. Consequently, apoptosis can be activated following disruption of the SAHF by treatment with a histone deacetylase inhibitor [43]. However, the outcome of inhibiting the SAHF may be context specific. For example, disrupting a key histone methyltransferase Suv39h1 that contributes to the SAHF promotes tumor progression, particularly T cell lymphomagenesis, in a neuroblastoma Ras viral oncogene homolog (*N*-ras) transgenic mouse model [44].

Formation of the SAHF does not merely depend on the redistribution of repressive epigenetic marks, but requires key molecular and chromatin changes. Such a molecular change that promotes SAHF formation is activation the p16^INK4A^ retinoblastoma pathway and, therefore, strongly correlates with cell cycle arrest [36,45,46]. However, several studies have challenged the requirement of the SAHF for cellular senescence and demonstrated that it is not universal among all senescent cell types and senescence-inducing stimuli. For instance, the SAHF are most prominently observed during oncogene-induced senescence, but also form during replicative senescence [36,47]. However, fibroblasts derived from patients afflicted with Hutchinson-Gilford progeria syndrome (HGPS) that are intrinsically prone to cellular senescence do not develop features of the SAHF [47,48]. Studies have used high-throughput whole-genome conformation capture methods to interrogate formation of the SAHF during cellular senescence [49,50]. Following senescence induction, chromatin become reorganized causing heterochromatin to dissociate from the nuclear periphery. Therefore, the heterochromatin that is incorporated into the SAHF is not newly formed, but a result of heterochromatin redistribution [36]. The dissociation of heterochromatin from the nuclear periphery is mediated by loss of lamin B1, a nuclear lamin protein that associates with lamina-associated domains within heterochromatin [46,51]. Interestingly, lamin B1 abundance is decreased in multiple senescent cell types and in mice tissue following irradiation-induced senescence [52]. Lamin B1 appears to be downregulated at the transcriptional level during the induction of cellular senescence, and silencing lamin B1 is also sufficient to induce cellular senescence [53]. In addition to being downregulated, lamin B1 preferentially binds H3K27me3-enriched sites that are associated with transcriptional repression and the SAHF during cellular senescence [46]. Loss of lamin B1 also appears to cause a redistribution of histone marks, including an enrichment in H3K27me3 and H3K4me marks within lamin B1-associated domains and depletion in H3K27me3 marks in enhancers and genes. Interestingly, the particular genes lacking H3K27me3 marks that were upregulated were found to be senescence-related genes, including SASP genes [51].

Following loss of lamin B1, heterochromatin undergoes the subsequent steps of decondensation and spatial clustering to form the SAHF [50]. Although the mechanism behind spatial heterochromatin clustering to form the SAHF remains unknown, the identification of heterochromatin decondensation during cellular senescence is consistent with the progressive loss of heterochromatin that is observed during aging and in diseases of premature aging, including HGPS and Werner syndrome [54,55,56]. It is also unknown if the progressive loss of heterochromatin is linked to the reduction in histone biosynthesis that appears to influence the redistribution in epigenetic marks during replicative senescence [57]. Such deregulations in heterochromatin structure may allow for expression of retrotransposable elements that have been shown to drive genomic instability during cellular senescence and tissue aging [58,59,60].

### 2.2. Senescence-Associated Distention of Satellites Is a Senescence-Associated Heterochromatic Foci -Independent Epigenetic Change

Epigenetic changes are not limited to facultative heterochromatin regions that form the SAHF. The SAHF are actually distinct from constitutive heterochromatin that are present in telomeres and pericentromeres [61]. However, pericentric satellite DNA has been shown to undergo a dramatic decondendation during cellular senescence, independently from SAHF formation [58]. This nuclear phenotype has been termed senescence-associated distention of satellites (SADS) and appears to be exclusively formed in senescent cells and not in non-senescent cells and cancer cell lines [62]. Unlike SAHF formation, SADS formation is conserved among senescent cell types and senescence-inducing stimuli, including HGPS fibroblasts. Additionally, SADS formation has been identified in vivo and suspected to promote tissue aging [47,63]. Attempts to identify the role for SADS formation showed that it is an early event during senescence induction and precedes other nuclear changes, including nuclear enlargement and SAHF formation [62,64]. Additionally, SADS formation may be linked to hypomethylation and expression of pericentric satellite DNA that has been observed during cellular senescence [65,66]. However, the exact function of SADS during cellular senescence remains unknown [62]. Interestingly, pericentric satellite transcripts can promote mitotic errors and genomic instability, leading to the induction of cellular senescence. This particular study showed that sirtuin-6 (SIRT6) prevents these genomic stressors by silencing pericentric satellite transcripts and protects against cellular senescence [67]. It is possible that the expression of pericentric satellite transcripts is an early event during senescence induction that promotes genomic instability to help activate cell cycle arrest.

## 3. Epigenetic Effectors of Cellular Senescence

### 3.1. The Senescence-Associated Heterochromatic Foci and High Mobility Group Proteins Cooperate for the Senescence Phenotype

The SAHF appears to function in concert with and, in some cases, rely on other epigenetic effectors during cellular senescence. Key examples are proteins of the high mobility group (HMG) family, particularly HMGA1 and HMGB2. The HMG proteins are non-histone chromatin-binding proteins that remodel chromatin architecture, resulting in altered gene expression [68]. The HMGA proteins consist of HMGA1 and HMGA2. These have been shown to accumulate in the chromatin of senescent cells, binding the same site and causing displacement of linker histone H1, and structurally support the SAHF [69]. In this manner, HMGA proteins cooperate with p16^INK4A^ to maintain the proliferative arrest of the senescent cells [70]. This tumor suppressive function of the HMGA proteins was surprising considering that the HMGA proteins were previously associated with gene activation under proliferative states, such as embryogenesis and cancer [68,71]. Unlike members of the HMGA proteins, studies have found members of the HMGB proteins to have dissimilar mechanisms of action during cellular senescence. The HMGB1 protein is secreted in a p53-dependent manner and functions as an extracellular alarmin that activates nuclear factor-κB (NF-κB) and proinflammatory signaling pathways [72]. The HMGB2 protein also has a proinflammatory role during cellular senescence, but through a mechanism that is distinct from HMGB1. The HMGB2 protein binds the loci of key SASP genes and prevents their incorporation into transcriptionally repressive SAHF regions, providing a chromatin landscape that is conducive for SASP gene expression. Interestingly, inhibition of HMGB2 limits the SASP without affecting the senescence proliferative arrest. These results indicate that the deleterious, pro-tumorigenic, SASP can be uncoupled from the SAHF, which is associated with the beneficial tumor suppressive proliferative arrest of the senescent cells [73].

### 3.2. Epigenetic Regulators of the Senescence Associated Secretory Phenotype

Similar to what has been found following inhibition of HMGB2, several studies have found it is possible to target epigenetic mechanisms that specifically drive the SASP, as a therapeutic means [73]. It has become clear that senescent cells can transcriptionally regulate the SASP through other epigenetic mechanisms and effectors in addition to the HMGB2. For instance, super-enhancers are formed adjacent to key SASP genes following remodeling in the enhancer chromatin landscape during cellular senescence. These super-enhancers are enriched in H3K27 acetylation and recruit the bromodomain and extra-terminal domain (BET) protein BRD4 to promote SASP gene expression. An important aspect of this study is that inhibition of BRD4 suppressed SASP gene expression without affecting the proliferative arrest of the senescent cells. Moreover, BRD4 inhibition was also shown to have therapeutic efficacy against senescent cells in vivo by suppressing the SASP along with its subsequent immune surveillance response [74]. Expression of the SASP is also directly regulated by the histone variant macroH2A1, a component of the SAHF. Interestingly, macroH2A1 was found to be not only required for SASP gene expression, but also DNA damage response signaling during cellular senescence. This led to the identification of a negative feedback loop whereby macroH2A1 activates DNA damage response signaling that leads to the removal of macroH2A1 from SASP gene loci, resulting in a dampened SASP [75]. Expression of the SASP was also found to be negatively regulated by sirtuin-1 (SIRT1) in a direct manner. In the event of SIRT1 knockdown or decreased expression, which is observed during cellular senescence, this study found acetylation of H3K9 and H4K16 is increased at the promoters of IL-6 and IL-8, resulting in the transcriptional activation of these cytokines. Therefore, SIRT1 was proposed to deacetylate H3K9 and H4K16 in the promoter regions of the SASP factors IL-6 and IL-8, causing their transcriptional repression under normal non-senescent conditions [76].

Epigenetic factors can also activate pro-inflammatory signaling that underlies activation of the SASP, as opposed to directly modifying SASP gene loci as discussed above. This was found in the case of the methyltransferase mixed-lineage leukemia 1 (MLL1) during cellular senescence. The MLL1 protein activates the expression of proliferation-related cell cycle genes during senescence induction, causing hyper-replicative stress that triggers the DNA damage response. This results in activation of the NF-κB pro-inflammatory signaling pathway that drives SASP gene expression. Importantly, this study showed that inhibiting MLL1 suppresses SASP gene expression without causing senescent cells to escape the proliferative arrest, indicating the therapeutic potential to intervene with MLL1. This point was further highlighted by the fact that MLL1 inhibition was able to suppress the SASP and inflammation associated with cancer in vivo [77]. Separate from activating proinflammatory signaling, the DNA damage response has been shown to induce epigenetic changes that activate SASP gene expression during cellular senescence. Mechanistically, the DNA damage response activates proteasomal degradation of the histone methyltransferases G9a and G9a-like protein (GLP). This results in a decrease in transcriptionally repressive H3K9 dimethylation marks at key SASP gene promoters, leading to enhanced gene expression [78]. Interestingly, the DNA damage response can also become activated and promote the SASP following chromatin remodeling, independent of physical breaks in the DNA. In particular, inhibition of histone deacetylase 1 (HDAC1), which leads to hyperacetylation of histone and non-histone proteins, has been shown to increase the expression of a key proinflammatory SASP factor, osteopontin. Consequently, HDAC1 inhibition promotes a protumorigenic microenvironment and tumor growth in vivo [79].

## 4. Conclusions

**Figure 1:**
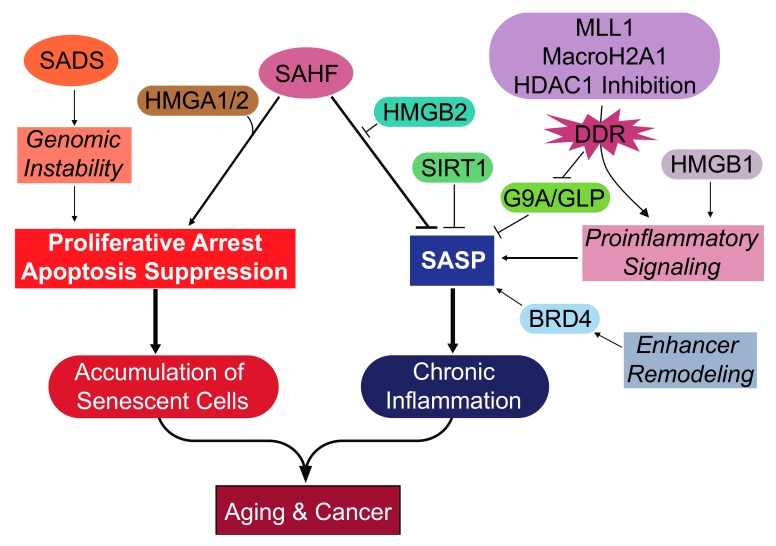
Overview of the epigenetic events and effectors during cellular senescence. Key epigenetic changes are development of the SADS and SAHF. The formation of SADS is an early epigenetic change that may aid in the growth arrest by promoting genomic instability. The SAHF collaborates with other epigenetic effectors and has several functions. The HMGA proteins structurally support the SAHF and aid in the repression of proliferation-promoting genes, resulting in the proliferative arrest. The HMGB2 protein prevents the incorporation of SASP gene loci into the transcriptionally repressive SAHF, thereby promoting the SASP. Transcriptionally repressive marks to SASP gene loci can be made by SIRT1 and G9a/GLP. The expression of the SASP is promoted by proinflammatory signaling mediated by MLL1, macroH2A1, HDAC inhibition, and HMGB1. Senescent cells also undergo remodeling in the enhancer landscape that promotes the expression of the SASP, which is mediated by BRD4. These epigenetic mechanisms support the proliferative arrest of senescent cells, which accumulate and impair tissue function, leading to aging. Another long-term deleterious effect of senescent cells is the SASP that activates chronic inflammation and promotes both aging and cancer. SAHF: Senescence-associated heterochromatic foci; SADS: Senescence-associated distention of satellites; SASP: Senescence-associated secretory phenotype; HMGA1/2: High mobility group A 1/2; HMGB1/2: High mobility group B 1/2; MLL1: Mixed-lineage leukemia 1; BRD4: Bromodomain-containing 4; SIRT1: Sirtuin-1; HDAC1: Histone deacetylase 1; GLP: G9a-like protein; DDR: DNA damage response.

Senescent cells undergo distinct epigenetic changes that serve several effector functions, which are summarized in Figure 1 and Table 1. Such examples are the SAHF and SADS, which are formed following the remodeling in facultative and constitutive heterochromatin, respectively. The SAHF are formed following exit from the cell cycle and aid in senescence induction by stabilizing the proliferative arrest [36,45]. The SAHF may also ensure cell survival during senescence induction by suppressing apoptosis [43]. It appears that senescent cells develop SADS prior to SAHF formation, although the link between these two epigenetic events is not completely understood. A possible role for the SADS may be to upregulate the expression of pericentric satellite transcripts that promote genomic instability and activate the DNA damage response to arrest proliferation [62]. Thus, SADS and SAHF formation appear to serve tumor suppressive roles of senescent cells. However, it may be beneficial to augment the function of the SAHF during cellular senescence. For instance, the SAHF may be augmented to silence SASP genes in addition to proliferation-related genes following inhibition of HMGB2 [73]. It is also possible that inhibition of HMGB1 along with HMGB2 may yield a greater suppression of the SASP, considering the distinct pro-inflammatory role of HMGB1 [72]. In support of this notion, a small molecule inhibitor of HMGB1 and HMGB2 was shown to have anti-inflammatory effects in the context of microglia-mediated neuroinflammation [80]. However, the effects of such a compound remain to be determined under different contexts.

Several epigenetic mechanisms mediate the SASP during cellular senescence. Interestingly, many of the epigenetic effectors associated with these mechanisms have previously been shown to play a role in tumorigenesis and may lie at the interface between cancer and aging. For instance, many of the HMG proteins are overexpressed and support transcriptional reprogramming in different cancer cell types, and are associated with a poor prognosis [68,81,82]. The MLL1 protein undergoes chromosomal translocations and aberrantly upregulates genes related to development and the cell cycle, promoting tumorigenesis of several leukemias [83,84]. The BRD4 protein plays several roles in cancer by upregulating oncogenes, such as c-myc, and genes related to proliferation, apoptosis suppression, and inflammation [85,86]. Clinical trials using inhibitors against BRD4 and MLL1 in cancer are still underway and, if successful, will be interesting to determine if attenuation of the protumorigenic effects of senescent cells is part of the success, such as inhibition of the SASP. Additionally, the targeting of senescent cells may be enhanced by combining epigenetic inhibitors with senolytic compounds, which has yet to be explored. Moreover, these types of studies will raise the possibility of targeting epigenetic mechanisms of cellular senescence not only to treat cancer, but also pathologic states associated with tissue aging.

## Figures and Tables

**Table 1:** Epigenetic changes and effectors of cellular senescence.

Heterochromatin Modification or Activated Effector	Mode of Cellular Senescence	Function during Cellular Senescence	Cell Type	Reference
SAHF	Replicative Oncogene	Proliferative Arrest	Human Fibroblasts	[36,37,61]
SADS	Replicative Oncogene HGPS	Not Defined	Human Fibroblasts	[58,62]
Loss of Lamin B1	Replicative Oncogene HGPS Genotoxic Stress: Irradiation	Proliferative Arrest	Human Fibroblasts	[51,52,53]
HMGA1/2	Replicative Oncogene	Proliferative Arrest	Human Fibroblasts	[70]
HMGB1	Replicative Oncogene Genotoxic stress: Irradiation	SASP Activation	Human Fibroblasts	[72]
HMGB2	Oncogene	SASP Activation	Human Fibroblasts	[73]
HDAC1	Genotoxic stress: Bleomycin HDAC Inhibitors: Sodium butyrate and Trichostatin A	SASP Activation	Human Fibroblasts	[79]
Enhancer Remodeling: BRD4	Oncogene Genotoxic stress: Etoposide	SASP Activation	Human Fibroblasts	[74]
MLL1	Oncogene Genotoxic stress: Etoposide	SASP Activation	Human Fibroblasts MCF7 human breast cancer cell line	[77]
MacroH2A1	Oncogene	SASP Activation	Human Fibroblasts	[75]
G9a/GLP	Replicative Oncogene	SASP Inhibition	Human Fibroblasts	[78]
SIRT1	Genotoxic stress: Irradiation	SASP Inhibition	Human Fibroblasts	[76]

HGPS: Hutchinson–Gilford progeria syndrome.
